# Troxerutin protects against DHT-induced polycystic ovary syndrome in rats

**DOI:** 10.1186/s13048-020-00701-z

**Published:** 2020-09-13

**Authors:** Zixuan Gao, Xiaochen Ma, Jing Liu, Yuhang Ge, Lei Wang, Ping Fu, Zhian Liu, Ruiqin Yao, Xiaonan Yan

**Affiliations:** 1 Department of Histology and Embryology, Xuzhou Medical University, Xuzhou, 221009 PR China; 2 Department of Cell Biology and Neurobiology, Xuzhou Key Laboratory of Neurobiology, Jiangsu Key Laboratory of New Drug Research and Clinical Pharmacy, Xuzhou Medical University, 209 Tongshan Road, Xuzhou, 221009 PR China; 3 Clinical Center of Reproductive Medicine, Xuzhou Central Hospital, 199 Jiefang South Road, Xuzhou, 221000 PR China; 4 Department of Human Anatomy, Xuzhou Medical University, Xuzhou, 221009 PR China

**Keywords:** PCOS, Troxerutin, GnRH, LH, Neurotransmitter

## Abstract

The exact pathogenesis of polycystic ovary syndrome (PCOS), the most common neuroendocrine disorder in women of reproductive age, has not been fully elucidated. Recent studies suggested that chronic inflammation and neurotransmitter disorder involved in the progress of PCOS. Troxerutin, a natural flavonoid, was reported to possess neuroprotective effect in several disease models by inhibiting inflammation or enhancing neurotrophic factor. In this study, we investigated the possible protective effect and mechanism of troxerutin in a dihydrotestosterone (DHT)-induced rat model of PCOS. The PCOS rat models were treated with troxerutin at a dose of 150 mg/kg or 300 mg/kg for up to 4 weeks. Results showed that 300 mg/kg troxerutin significantly decreased the body weight gain and improved the pathological changes of ovary induced by DHT. Meanwhile, the elevated gonadotrophin-releasing hormone (GnRH), gonadotrophin and testosterone in the serum of PCOS rats were reduced with the treatment of troxerutin. The expression of kisspeptin and NKB in arcuate nucleus and their receptors kiss1r and NK3r in GnRH positive neurons of median eminence were markedly decreased in troxerutin-treated rats. Of note, the GnRH inhibitory regulator GABA and stimulatory regulator glutamate were also restored to the normal level by troxerutin. The present study indicated that troxerutin may exhibit a protective effect in PCOS rat model via regulating neurotransmitter release.

## Introduction

Polycystic ovary syndrome (PCOS) is a reproductive endocrinopathy with the prevalence estimated to be 6% ~ 20%(depending on the different diagnostic criteria used), making it the most common endocrine condition in women of reproductive age [1]. The diagnostic features of PCOS include androgen excess, ovulatory dysfunction and polycystic ovaries [1]. Obesity is present in 30–60% of PCOS patients, depending on the country of origin [2, 3]. The most consistent biochemical abnormality in women with PCOS is hyper-secretion of androgen, elevated serum luteinizing hormone (LH) levels and low to normal serum follicle stimulating hormone (FSH) levels [4, 5] . Animal models that reflect PCOS features are crucial resources to investigate this syndrome. A chronically 5α-dihydrotestosterone (DHT)-treated rat model closely mimics the human PCOS phenotype and is a suitable model for investigations about PCOS.

The hypothalamus-pituitary-gonad (HPG) axis plays a central role in the exquisite neuroendocrine regulation of reproduction. Hypothalamic secretion of gonadotrophin-releasing hormone (GnRH), has been robustly established as the key pathway that controls reproductive function [6]. Axons of GnRH neurons protrude into the median eminence and release GnRH, which via hypophyseal portal system, enters anterior pituitary. Anterior pituitary, the primary target of GnRH, responds to stimulation by increasing the secretion of LH and FSH, which in turn lead to steroid production from the ovaries and stimulate follicle genesis and ovulation [7]. Thus, GnRH is known as the master hormone and provides the final common output of the network that regulates reproductive function.

GnRH is influenced by extrinsic factors such as neurotransmitters and neuropeptides, any alteration of the GnRH regulatory neurotransmitters, such as inhibitory gamma-aminobutyric acid(GABA) and stimulatory glutamate (Glu), may result in reproductive endocrine dysfunction [8]. Actually, the chronic anovulation characteristic of PCOS is attributed to increased central GnRH drive and resulting gonadotrophin aberrations, which likely result from the cumulative effect of altered GnRH stimulatory and inhibitory neurotransmitter in hypothalamus and pituitary gland [9]. Kisspeptin and subsequently discovered neurkinin B (NKB), two novel GnRH regulatory neuropeptides proved to be essential for normal GnRH secretion in humans, have come under intense spotlight in the last decade [10, 11] . The related discovery of kisspeptin−/ neurokinin B−/ dynorphin-(KNDy) pathway has further strengthened the understanding of GnRH secretion modulation [12] . KNDy neurons residing in the arcuate nucleus region of rodents co-express kisspeptin, NKB and DYN [12, 13]. NKB and kisspeptin derived from KNDy stimulate the release of GnRH by binding Kisspeptin 1 receptor (Kiss1r) and neurokinin 3 receptor (NK3r) expressed on GnRH neurons [14, 15].

Troxerutin, a trihydroxyethylated derivative of the naturally occurring plant bioflavonoid rutin, also called vitamin P4. It has been widely identified in plants consumed as part of our daily diet, such as tea, coffee, cereals, and a variety of fruits and vegetables, and exerts multifarious biological activities, such as anti-oxidant and anti-inflammatory effects and exerts beneficial effects on animal models of different central nervous system diseases [16, 17]. Troxertuin attenuated cognitive deficit and oxidative stress in mouse brain through decreasing reactive oxygen species [18],inhibited hippocampal neuron apoptosis in a rat model of Alzheimer’s disease [19]. Troxerutin inhibited cyclin-dependent kinase 1 expression, enhanced type 1 protein phosphatase α dephosphorylation and abolished MEK/ERK1/2/C/EBP β activation, which subsequently reversed the memory impairment in the domoic acid-treated mice [20], and exhibited neuroprotective potential in 6-OHDA rat model of Parkinson’s disease through mitigation of apoptosis,astrogliosis and oxidative stress [21], prevented the harmful effects of maternal high-fat diet on their offspring through inhibition of pro-inflammatory cytokines and elevation of brain-derived neurotrophic factor levels [22], significantly promoted nerve growth factor (NGF) mRNA expression and attenuated cognitive impairment and brain oxidative stress through the activation of NGF/TrkA signaling pathway in D-gal-injected mice [23]. However, there was no evidence that troxerutin had any effect on IGF-1 so far. Since the hypothalamus plays an important role in reproduction, we supposed that troxerutin may have a protective effect against reproductive neuroendocrine dysfunction in PCOS. This study aimed to address this issue and preliminarily investigate the potential mechanism underlying its action.

## Materials and methods

### Animals and troxerutin administration

Postnatal 14 days female SD rats were purchased from the Breeder center of Rodents (Jinan, China). The rats were housed under controlled lighting (12 h light, 12 h darkness, lights were turned on at 08:00 and turned off at 20:00) and temperature (24 °C) with free access to food and water for a week of acclimatization. All experimental protocols used in this study were approved by the Animal Care and Ethical Committee of Xuzhou medical University. Postnatal 21 days rats weighing approximately 50-55 g, were randomly divided into four groups: sham group (each rat was implanted with a tube without DHT), PCOS group (each rat was implanted with a silastic tube filled with 5α- DHT at dose of 7.5 mg under anesthesia), PCOS+ 150 mg/kg troxerutin group and PCOS+ 300 mg/kg troxerutin group (PCOS rat with intraperitoneal injection of 150 mg/kg or 300 mg/kg troxerutin daily during the 4 weeks after implantation). Due to the high water-solubility of troxerutin, the gastrointestinal system can absorb it well without toxicity. Troxerutin interacts with tRNA by external binding manner with low binding affinity, and also binds with DNA through groove binding manner. Sham and PCOS groups were injected daily with equivalent 0.9% saline which was used to dissolve troxerutin. DHT was procured from sigma, St. Louis, Mo and troxerutin (purity > 95%) was from Baoji Fangsheng Biotechnology Co., Ltd., Baoji, China. The rats were housed individually after surgery.

### Blood and tissue sampling

At the terminal of the study, rats were decapitated and trunk blood was collected and centrifuged at 3000 rpm for 15 min. Plasma was separated and stored at − 80 °C until analyzed for biochemical and hormonal analysis. Ovaries were cleaned in saline and managed fat free. Ovaries were fixed in 10% buffered formalin for 48 h and paraffin-embedded. Paraffin-embedded tissue sections were de-waxed, sectioned (6 μm) and stained with hematoxylin and eosin (H&E). Numbers of follicles were classified and counted in every 40th ovarian section for 10 ovaries per group. Follicles were counted according to the following definition. Primordial follicle was surrounded by a single layer of squamous granulosa cells. Primary follicle contained a single layer of cuboidal granulosa cells. Secondary follicle contained two or more layers of granusola cells, but no antral space in the granulosa layer. Antral follicle displayed multi-layers of granusola cells with the presence of follicular antrum.

### Vagina smears and estrous cycles

Vaginal smears were taken daily at 09:00 am from the 18th to the 28th day after the first day of DHT treatment,which were the terminal 10 days of the study. Microscopic analysis (BX41, Olympus) was carried out to determine the stage of the estrous cycle by the predominant cell types in vaginal smears following methylene blue staining (Solarbio, Beijing). Proestrus consists of enlarged,round,nucleated epithelical cells; estrus consists of a large number of cornified squamous epithelical cells; metestrus consists of two kinds of cells: epithelial cells and leukocytes with approximately same amount; and diestrus is characterized by a small amount of cells with the predominance of leukocytes.

### Body weight, biochemical and western blot analysis

Body weight was measured every week after implantation for a total of 4 weeks.

The serum concentration of LH, FSH and testosterone were measured via Enzyme Linked Immuno Sorbent Assay (ELISA) with the help of commercial kits (ELISA kit, CUSABIO, Inc., Wuhan, China) and the procedure was followed as given in the kit catalog. The concentration of LH, FSH and testosterone was estimated by the standard curve.

To identify the hypothalamic GnRH status, hypothalamus was dissected out. The rat brain was carefully taken out and temporarily put on ice with the ventral surface upward. Thalamus is the ellipsoidal part at the center of the ventral brain. With the middle of tuber cinereum and optic chiasma as the center, prechiasmal border as the anterior edge, postmammillary border as the posterior edge, hypothalamus (4x4x2mm) was carefully dissected out. Hypothalamus tissue were homogenized to obtain protein samples. Then the protein (25 μg) was separated by SDS-PAGE and transferred to membranes. The membranes were incubated with mouse monoclonal anti-GnRH antibody (1:1000, MAB5456-C, Millipore, Billerica, MA) and mouse anti-GAPDH antibody (1:20000, Proteintech, Chicago, USA). After being washed with TBST three times, the membranes were incubated with IRDye-labeled secondary antibodies in TBST for 2 h. The bands on the membrane were scanned with an Odyssey infrared scanner (LI-COR Biosciences, Lincoln, NE, USA) and the density of the bands was analyzed with ImageJ software.

### Chromatographic analysis

Chromatographic analysis was carried out to evaluate the levels of two kinds of GnRH regulatory neurotransmitters GABA and glutamate in the brain/pituitary. Weigh the brain and add in the mix of methanol and water (1:1) at the ratio of 100 mg/ml to prepare homogenate. The brain homogenate was centrifuged at 12000 rpm for 30 min at 4 °C. The mix of supernate and 80 μL acetonitrile were centrifuged at 12000 rpm for 30 min at 4 °C. The new supernate was derivatized, mixed with 80 μL borate saline buffer, 160 μL water and 80 μL F-moc, filtered by 0.45 μm filtration membrane and immersed in 40 °C bath for 5 min. Then, the samples were used for the measurement of GABA and glutamate using chromatographic analysis. All standard biomarkers used for identification purpose in chromatographic studies were procured from Zhongke Co, Ltd., Beijing, China. High-performance liquid chromatography (HPLC) was performed on a Waters e2695 system (Waters, USA) equipped with a hypersil ODS column (Elite, Dalian, China). The mobile phase A was a mix of natrium aceticum (pH 4.8),water and tetrahydrofuran with the ratio of 410:85:5, and mobile phase B was pure acetonitrile. Flow rate was 1 ml/min. The column temperature was set at 30 °C. Samples were detected at the wavelength of 265 nm. Injection volume was 20 μL.

### Immunofluorescent staining

After intracardiac perfusion with normal saline followed by fixation with 4% cold paraformaldehyde (PFA), the rat brains were separated and postfixed in 4% PFA for 6 h at 4 °C. Then the brains were incubated in 30% sucrose- 100 mM sodium phosphate buffer (pH 7.4) for 48 h at 4 °C. Serial coronal sections (20 μm) were made from the bregma anterior-posterior − 2.0 mm to − 3.30 mm. Totally 60 sections were collected per brain. At least three sections per brain were selected for NKB, Kisspeptin, GnRH/NK3R and GnRH/Kiss1r immunofluorescent staining, respectively.

For immunofluorescence, the primary antibodies rabbit anti-NKB (1:1000, Novus biologicals, NB300–201), rabbit anti-kisspeptin (1:1000, H-048-56, Phoenix Pharmaceuticals), rabbit anti-Kiss-1r (1:500, AKR-001, Alomone Labs), rabbit anti-NK3r(1:500, abx217136, Abbexa) and mouse anti-GnRH (1:1000, MAB5456-C, Millipore, Billerica, MA) were used. After incubating with the antibodies for 24 h at 4 °C, the sections were washed with PBS and then were treated with goat anti-mouse IgG (H + L) Alexa Fluor®555 or 488 -conjugated or goat anti-rabbit IgG (H + L) Alexa Fluor®488 (Invitrogen, Eugene, OR, USA) secondary antibodies. According to the manufacturer’s instructions, DAPI (Beyotime Biotechnology, Shanghai, China) was used to label nucleus. For negative controls, sections were incubated with PBS instead of the primary antibodies. Fluorescence images were captured using a Zeiss Axioskop 40 microscope (Carl Zeiss, Oberkochen, Germany). Image Pro-Plus 6.0 software was used for semi-quantitative measurement of immunofluorescent density. Values (three slides for each brain) of integral optical density (IOD) in individual cells represented the quantity of objective protein and were calculated using the following equation: Σ IOD/Σ DAPI.

### Statistical analysis

All statistical analyses were performed with SPSS software (version 16.0), and the data were analyzed using the one-way Analysis of Variance (ANOVA). The data were expressed as the mean ± s.e.m. Statistical significance was set at *P* < 0.05 for all tests.

## Results

### Troxerutin reduced body weight, improved abnormal ovarian morphology and function in PCOS rats

**Fig. 1:**
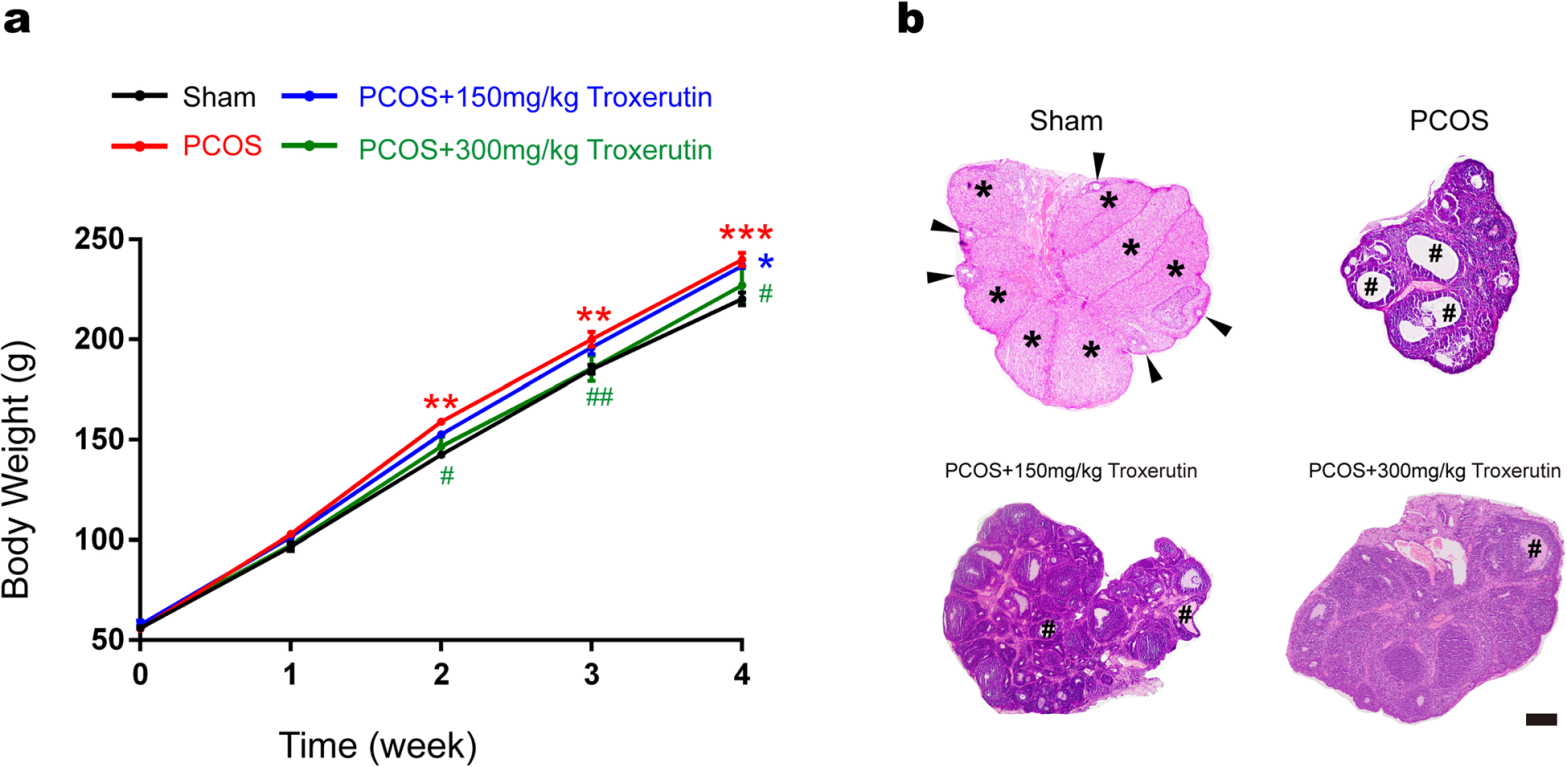
Troxerutin reduced body weight, improved abnormal ovarian morphology and function in PCOS rats. **a** Body weight **b** Hematoxylin and eosin staining of representative ovaries **(c)** Percentage of the number of follicles of total **d** Representative vagina smear and estrus cycle. For **a** and **c**, *P values* were determined by one-way ANOVA with Tukey’s multiple comparison test and data were presented as means±s.e.m. *n* = 12 rats per group. *P < 0.05, **P < 0.01, ***P < 0.001 vs. sham group; ^#^*P* < 0.05, ^##^*P* < 0.01 vs. PCOS group. For **b**, follicles in various stages of development were indicated by black triangles, corpora lutea were indicated by asterisks, while the cystic follicles were indicated by hashtags. Scale bar: 200 μm. For **d**, P,proestrus; E,estrus;M,metestrus;D,diestrus.Scale bar: 50 μm. Images were representative of three independent experiments with similar results

We first investigated whether troxerutin administration has any influence on body weight in DHT-induced PCOS rat models. The time course of troxerutin administration and evaluation of phenotypes were illustrated (Fig. 1). Body weight for individual animal was weighed weekly after DHT implantation for up to 4 weeks. Body weight at baseline and 1st week showed no significant difference among all groups. DHT-treated rats showed significantly higher body weight at 2nd (*P* < 0.01), 3rd(*P* < 0.01) and 4th (*P* < 0.001) week than the age-matched sham rats which denoted normal body weight, indicating that PCOS group have gained more body weight, hence a PCOS-like over weight feature. In comparison to PCOS group, treatment with troxerutin 150 mg/kg did not induce any significant changes. Nevertheless, the body weight of PCOS+ 300 mg/kg troxerutin group were markedly reduced as compared to that of PCOS group at 2nd (*P* < 0.05),3rd(*P* < 0.01) and 4th (*P* < 0.05) week, suggesting that troxerutin alleviated increased body weight in PCOS. Since ovarian histological change is another feature of PCOS, we also investigated whether troxerutin improved histological structure in rat ovaries. As demonstrated in Fig. 1b and c, normal ovarian histological features were observed in sham sections, which generally exhibited corpora lutea and follicles in various stages of development. In ovarian sections of PCOS rats, microscopic examination confirmed the absence of corpora lutea and the presence of cystic follicles,which were characterized by a large fluid-filled structure with an attenuated granulosa cell layer and a thickened theca internal cell layer. We furthered calculated the percentage of follicles of total and the results showed that PCOS rats exhibited significantly increased primordial and decreased primary follicles as compared to sham rats(*P* < 0.001; *P* < 0.001), and troxerutin 300 mg/kg inhibited the markedly low percentage of primary follicles in PCOS rats(*P* < 0.05). As compared to the multi-cystic follicles in ovaries of PCOS rats, the number of cystic follicles in troxerutin groups showed tendency toward decreasing. As Fig. 1.d demonstrated, sham group showed normal and stable estrous cycle, DHT induced cycle disruption in PCOS group, troxerutin administration 300 mg/kg in PCOS rat models prevented and substantially reversed the impaired estrous cycle. These findings suggest that troxerutin is a positive regulator of increased body weight,abnormal ovarian histological structure and ovary function in PCOS rats.

### Troxerutin reversed the abnormal serum levels of gonadotrophin and testosterone in PCOS rats

**Fig. 2:**
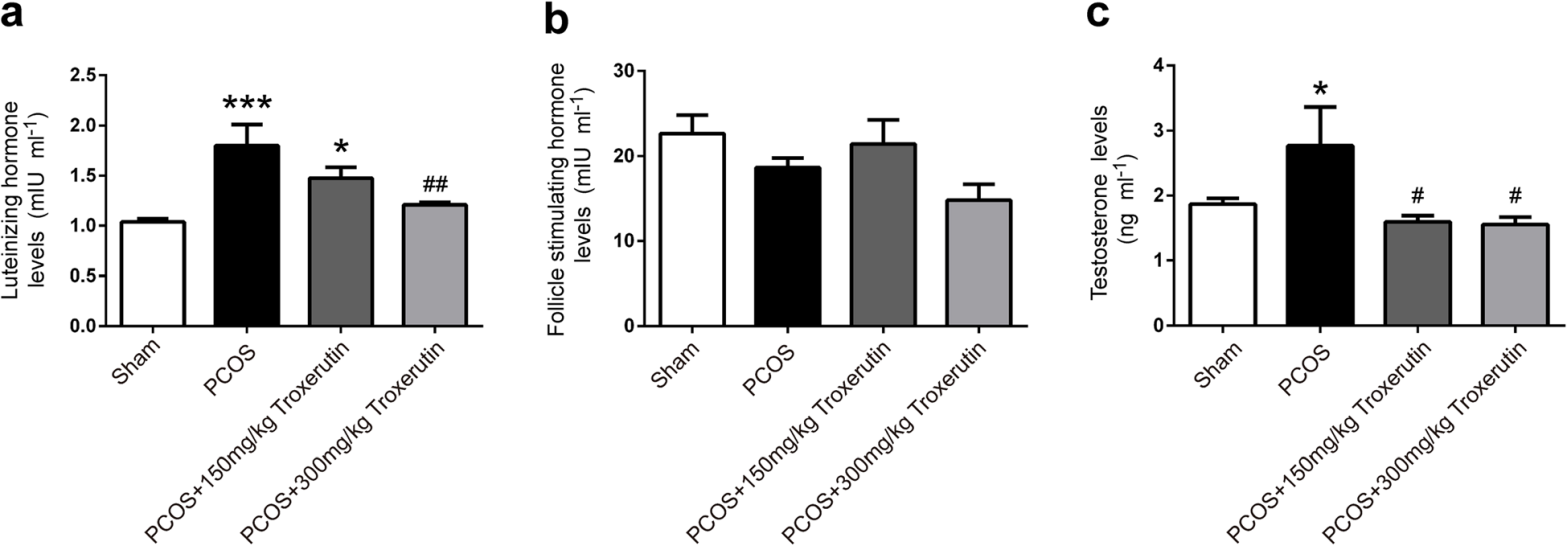
Troxerutin reversed the abnormal serum levels of gonadotrophin and testosterone in PCOS rats. **a** Serum LH levels **b** Serum FSH levels **c** Serum testosterone levels. *P values* were determined by one-way ANOVA with Tukey’s multiple comparison test and data were presented as means±s.e.m. *n* = 9 rats per group. *P < 0.05, ***P < 0.001 vs.sham group; ^#^*P* < 0.05, ^##^*P* < 0.01 vs. PCOS group

Serum LH, FSH and testosterone levels were measured at the fourth week by ELISA (Fig. 2). PCOS rats showed a significant increase in serum LH and testosterone levels compared to sham rats (*P* < 0.001; *P* < 0.05), which was in accordance with the characteristic LH and testosterone elevation in patients with PCOS. Though there was no significant difference in FSH levels among all four groups, administration of troxerutin to rats for 4 weeks significantly reversed the elevated serum LH (300 mg/kg; *P* < 0.01) and testosterone levels (both 150 mg/kg and 300 mg/kg; P < 0.05), suggesting that troxerutin has a beneficial effect on the aberrant gonadotrophin and testosterone in PCOS rats.

### Troxerutin inhibited the elevated GnRH levels in PCOS rats

**Fig. 3:**
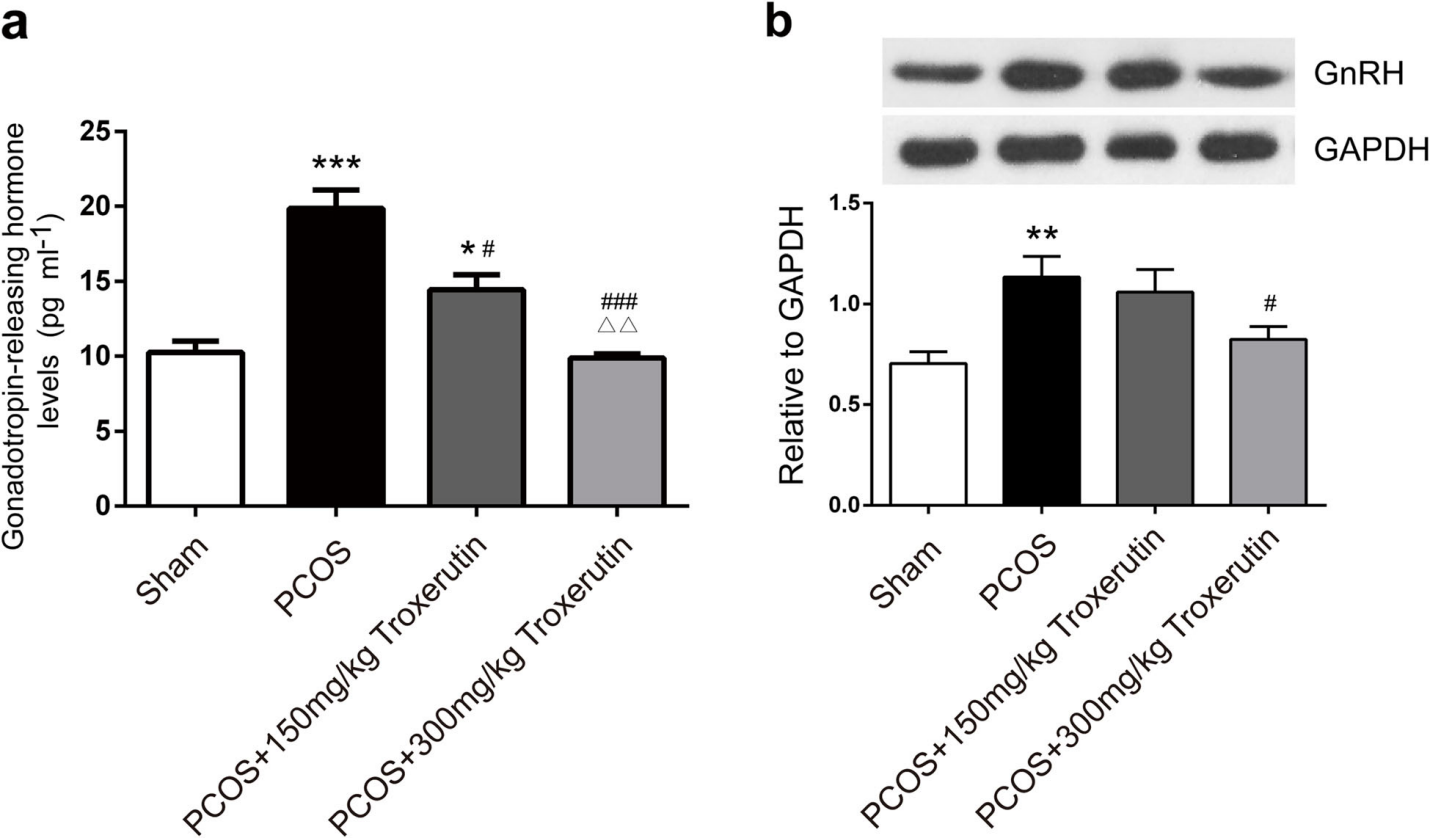
Troxerutin inhibited the elevated hypothalamic GnRH levels in PCOS rats. Hypothalamic status of GnRH. *P values* were determined by one-way ANOVA with Tukey’s multiple comparison test and data were presented as means±s.e.m. n = 9 rats per group. **P < 0.01; ^#^*P* < 0.05

The origin of LH and FSH alteration often times lies at hypothalamic GnRH level, which plays a pivotal role in stimulating pituitary release of gonadotrophin, thus we further detected the serum and hypothalamic-pituitary status of GnRH. As shown in Fig. 3, the western blotting results showed increased GnRH in hypothalamic-pituitary of the PCOS rats (*P* < 0.01), however, troxerutin 300 mg/kg markedly decreased the level of GnRH compared to the PCOS rats(*P* < 0.05; Fig. 3b).

### Troxerutin altered GnRH regulatory neurotransmitters in the hypothalamus of PCOS rats

**Fig. 4:**
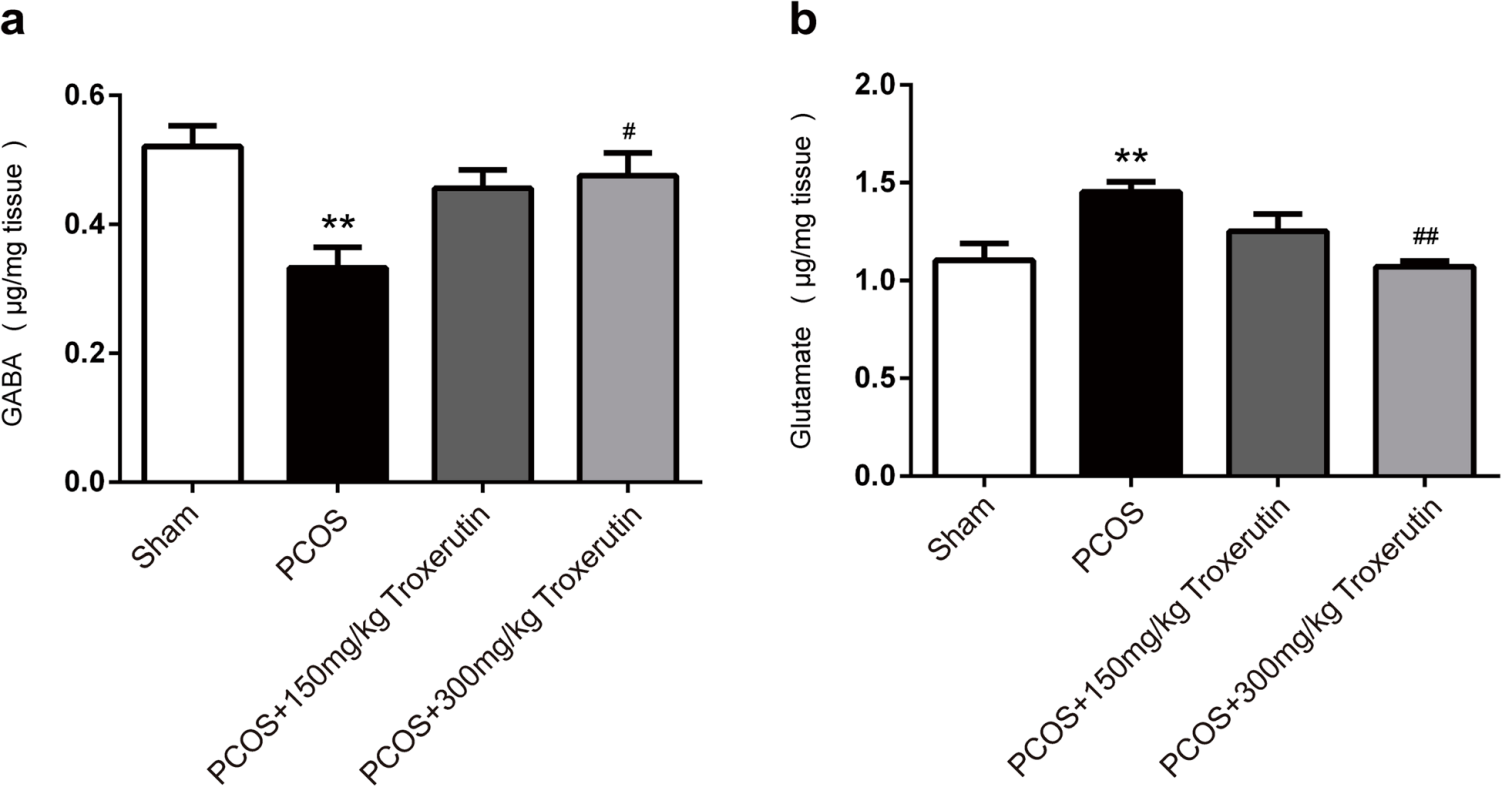
Troxerutin altered GnRH regulatory neurotransmitters in the hypothalamus of PCOS rats. **a** Hypothalamic levels of GnRH inhibitory GABA **b** Hypothalamic levels of GnRH stimulatory glutamate. *P values* were determined by one-way ANOVA with Tukey’s multiple comparison test and data were presented as means±s.e.m. n = 9 rats per group. **P < 0.01vs. sham group; ^#^*P* < 0.05, ^##^*P* < 0.01 vs. PCOS group

GnRH could be influenced by regulatory neurotransmitters such as the major inhibitory GABA and stimulatory Glu. As depicted in Fig. 4, neurotransmitter levels in the hypothalamus showed significant difference among certain groups. To be specific, PCOS rats exhibited markedly low GABA (*P* < 0.01) and high Glu (*P* < 0.01) compared to sham rats. Although no significant difference was observed between troxerutin 150 kg/mg group and PCOS group, administration of troxerutin 300 kg/mg to rats for 4 weeks successfully reversed the notably low GABA(*P* < 0.05) and high Glu (*P* < 0.01) levels in the hypothalamus compared to PCOS group.

### Troxerutin reduced the expression of Kisspepetin1/Kiss1r and Neurokinin B/NK3r in the hypothalamus

**Fig. 5:**
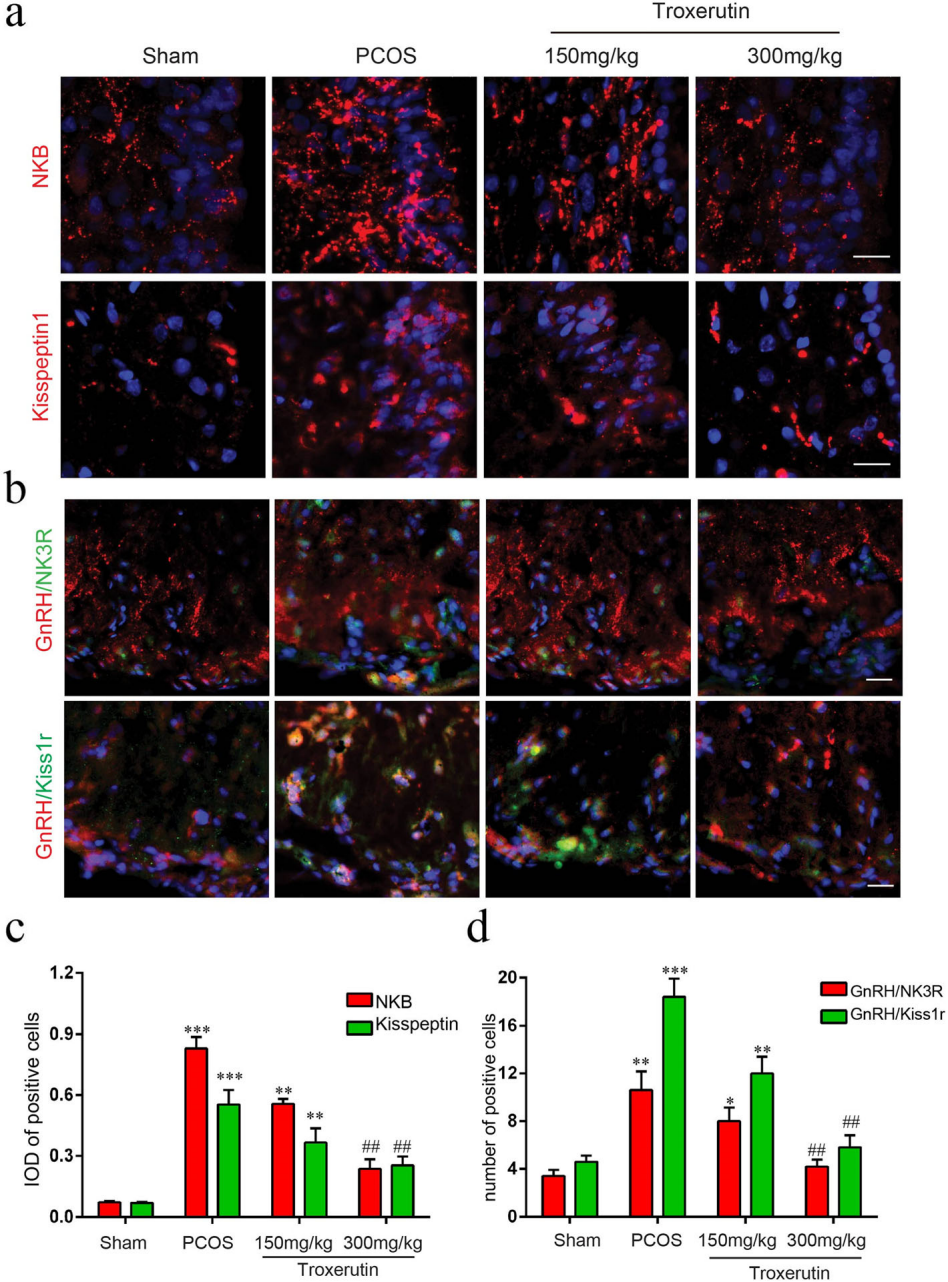
Troxerutin reduced the expression of Kisspepetin1 and Neurokinin B in the ARC. **a** Immunofluorescence photomicrograph showing that the expression of Kisspeptin1 and NKB in the arcuate nucleus (ARC). Scale bars:20 μm. **b** A lower magnification image showing the whole ARC and median eminence. Rectangular frame *a* and *b* represent ARC and median eminence (ME) analyzed in Fig. 5a and Fig. 6a, respectively. Scale bars:200 μm. **c** Quantitative analysis of integral optical density (IOD) of Kisspeptin1 and NKB in the ARC. *P values* were determined by one-way ANOVA with Tukey’s multiple comparison test and data were presented as means±s.e.m. *n* = 4 per group. **P < 0.01, ***P < 0.001vs. sham group; ^#^*P* < 0.05, ^##^*P* < 0.01 vs. PCOS group

**Fig. 6:**
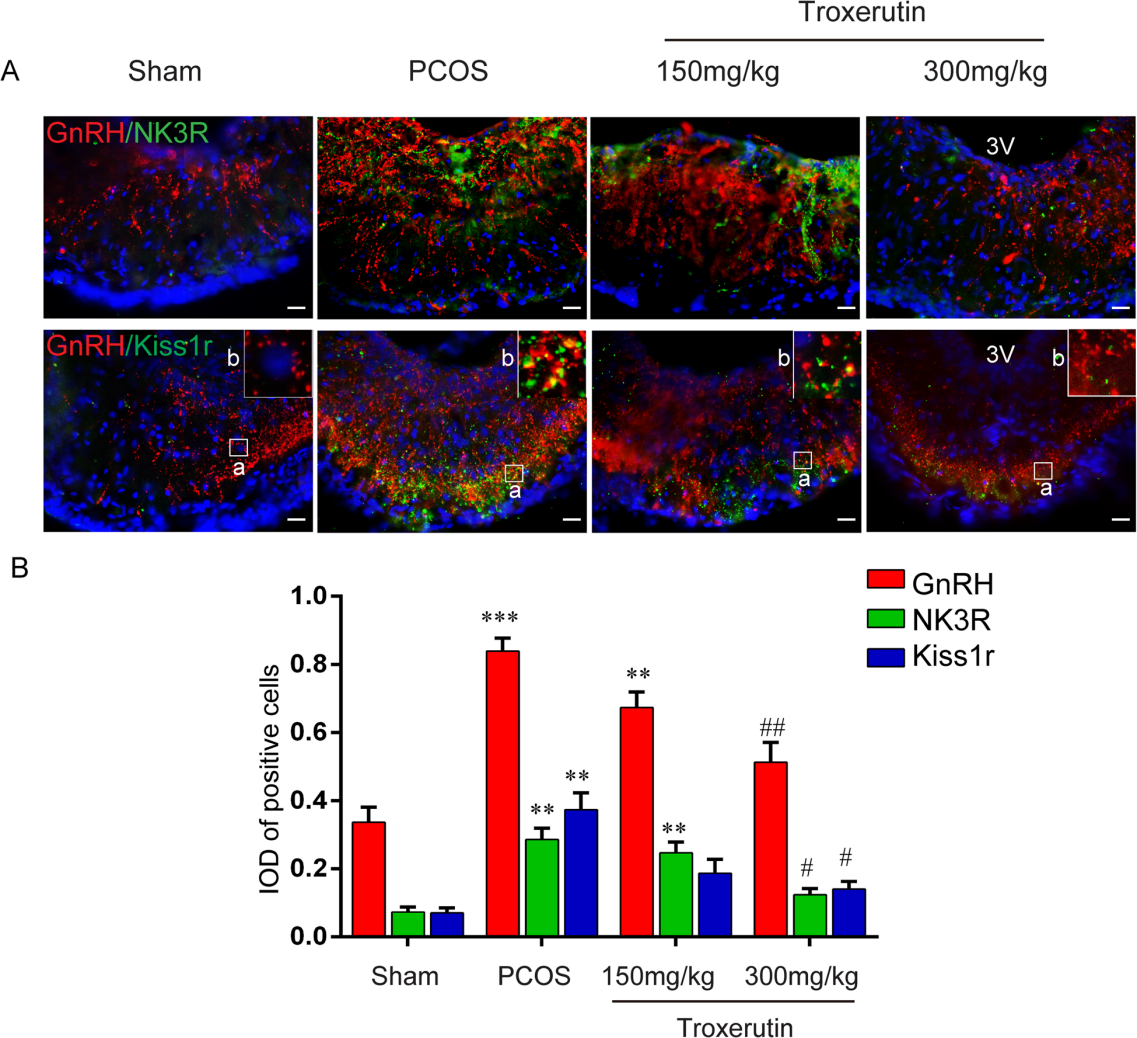
Troxerutin inhibited the expression of GnRH/Kiss1r and GnRH/NK3r in the ME. **a** Immunofluorescence photomicrograph showing that the expression of Kiss1r, NK3r and GnRH in the ME. Scale bar, 20 μm. Images on the frame *b* show higher magnification of the frame *a.* **b** Quantitative analysis of IOD of Kiss1r, NK3R and GnRH positive cells in the ME. *P values* were determined by one-way ANOVA with Tukey’s multiple comparison test and data were presented as means±s.e.m. n = 4 per group. **P < 0.01, ***P < 0.001vs. sham group; ^#^*P* < 0.05, ^##^*P* < 0.01 vs. PCOS group

Kisspeptin and NKB secreted from the KNDy neurons in ARC, are considered to be novel GnRH stimulatory neurotransmitters and stimulate expression of kisspeptin receptor (kiss1r) and neurokinin B receptor (NK3r) of GnRH neurons in the median eminence (ME). Here, we carried out immunofluorescence staining to observe whether troxerutin administration alters the expression of kisspeptin/ kiss1r and NKB/ NK3r in their respective regions (Fig. 5 and Fig. 6). The IOD of positive cells in the ARC were markedly increased in PCOS rats compared to sham rats (*P* < 0.001; *P* < 0.001), troxerutin 300 mg/kg treatment significantly reversed the increase of kisspeptin and NKB IOD induced by DHT (*P* < 0.05; *P* < 0.01). Meanwhile, the changes of the expression of kiss1r and NK3r in ME were also observed. The numbers of GnRH/kissr1/NK3r positive cells increased in the PCOS group compared to the sham group(P < 0.001; P < 0.01; P < 0.01), however, they were significantly decreased in the 300 mg/kg troxerutin-treated rat (P < 0.01; P < 0.05; P < 0.05) respectively.

## Discussion

PCOS is the most common endocrine condition affecting approximately 20% of reproductive -aged women. Though some ovulation induction agents, such as letrozole, clomiphene citrate and metformin, have been used to improve reproductive outcomes in clinic, the side effects of these drugs should not be ignored [24, 25]. Thus, numerous studies attempted to guide the development of new and effective therapies for PCOS. Recently, treatment based on traditional Chinese medicine and food ingredients, such as AF extract, soy isoflavones and crocetin, provided a novel therapeutic way for PCOS [26–28]. All these observations suggested rooms for improvement in PCOS therapies. Possession of beneficial effects on animal models of different central nervous system diseases made troxerutin, a rutin derivative, an attractive therapeutic method for us to investigate its possible effect on PCOS. Specifically, troxerutin inhibited cyclin-dependent kinase 1 expression, enhanced type 1 protein phosphatase α dephosphorylation and abolished MEK/ERK1/2/C/EBP β activation, which subsequently reversed the memory impairment in the Domoic acid-treated mice [20]. The neuroprotective potential of troxerutin in 6-OHDA rat model of Parkinson’s disease was through mitigation of apoptosis,astrogliosis, oxidative stress and part of its effect was dependent on PI3K/ERβ signaling [21]. Troxerutin and cerebroprotein hydrolysate injection acted as a neuroprotective agent against cerebral ischemia/reperfusion injury via anti-inflammation, anti-apoptosis and blood-brain barrier maintenance [29]. In the present study, we evaluated the possible effect of troxerutin for up to 4 weeks in a DHT-induced PCOS rat model, and two important findings were revealed. First, administering troxerutin 300 mg/kg exerted a beneficial effect of reducing body weight, the elevated levels of LH, testosterone and GnRH in PCOS rats and its effect was superior to troxerutin 150 mg/kg. Second, the potential mechanism behind the observed effect on increased body weight, polycystic ovaries, impaired estrous cycle and endocrine aberration involved altering GABA, glutamate, kisspeptin/kiss1r and NKB/NK3r in the hypothalamic pituitary region. To the best of our knowledge, this is the first study to investigate the possible effect of troxerutin on PCOS using a rat model and the potential mechanism of its protective effect may be via regulating GABA, glutamate, kisspeptin/kiss1r and NKB/NK3r in the brain/pituitary.

GnRH pulses stimulate the synthesis and secretion of LH and FSH from anterior pituitary. It is well known that although produced in the same cell named gonadotroph, LH and FSH synthesis is regulated by different frequency of GnRH pulses, with LH favored by fast pulse frequencies(> 1 pulse per hour) and FSH favored by slow pulse frequencies(< 1 pulse per 2–3 h). As for PCOS, a neuroendocrine hallmark is persistent and rapid GnRH pulses, which favor pituitary synthesis of LH and contribute to the increased LH levels [30]. LH is a stimulus for androgen synthesis, so the increased LH drive in turn caused the elevated androgens [31, 32]. Therefore, in terms of biochemical indicators, the most important and characteristic abnormality referring to PCOS is elevated LH, subsequently elevated testosterone and low to normal FSH levels in serum [4, 33]. Consistent with previous studies, PCOS rats displayed similar biochemical abnormality including markedly increased LH, concomitantly increased testosterone levels and non-significant FSH levels as compared to sham rats. Next, we observed the serum LH, testosterone and FSH levels in PCOS and troxerutin groups in order to determine whether troxerutin treatment has any therapeutic effect on PCOS. As the results revealed, troxerutin treatment indeed inhibited the elevated LH and testosterone levels without significant influence of FSH level. In addition, we further estimated the hypothalamic status of GnRH, which was significantly increased in PCOS rats. However, there was a significant troxerutin-caused (300 mg/kg) decline in hypothalamic GnRH. In regard to the serum hormone levels in rats, many previous studies have reported that LH were generally in the range of 1.5 ~ 3 mIU/ml [34, 35], FSH levels varied from 8mIU/ml to 20mIU/ml [26, 36] and testosterone varied from 0.2 to 10 ng/ml [34, 36–39], which were approximately consistent with the corresponding results in the present study. Conclusively, troxerutin treatment 300 kg/mg to rats for up to 4 weeks inhibited the hyperactive GnRH/LH system in PCOS rats.

Compelling evidence provided the strong support for brain being the culprit in PCOS [40, 41]. The secretion of GnRH from the brain is itself regulated by numerous upstream factors, such regulators include GABA, norepinephrine, dopamine, serotonin and glutamate,amongst others [42, 43]. While specific impairments in the brain were difficult to assess in humans, rat models greatly facilitated the identification of difference underpinning the hyperactive HPG axis in PCOS. Therefore, the representative and GnRH regulators of inhibitory GABA and stimulatory Glu were observed in the present study. In comparison to sham rats, PCOS rats showed a turnover of regulatory neurotransmitters with a significantly decreased GABA and increased Glu, which were both reversed by administering troxerutin 300 mg/kg to rats for up to 4 weeks. As far as the hypothalamic neurotransmitter levels were concerned, previous studies reported that hypothalamic GABA was in range of 12-20 μM/g and hypothalamic Glu was in range of 22-45 μM/g in rats,each approximately consistent with our findings after unit conversion. To date, one arcuate nucleus population of particular recent interest has been the kisspeptin/neurokinin B/dynorphin expressing ‘KNDy’ neurons,which synthesized and released GnRH stimulatory neurotransmitters kisspeptin and NKB. Protrusions of GnRH neurons in the ME express their receptors kiss1r and NK3r. In the present study, troxerutin decreased the expression of kisspeptin1 and NKB in the arcuate nucleus, and also the receptors kiss1r and NK3r in GnRH positive neurons of the ME.

## Conclusions

Taken above, these results indicated that the protection of troxerutin against PCOS partially might be due to its ability to regulate hypothalamic GABA, Glu, kisspeptin/kiss1r and NKB/NK3r. Although it remains determined whether this troxerutin-caused reversal is permanent, the present study highlighted its therapeutic potential for PCOS. More in depth research will be necessary to determine whether troxerutin targeted at hypothalamic neurotransmitters alone is a promising therapeutic approach for the treatment of PCOS.

## Data Availability

The datasets used and/or analyzed in the present study are available from the corresponding author on reasonable request.

## Abbrevations

ANOVA: Analysis of Variance
ARC: arcuate nucleus
DHT: Dihydrotestosterone
ELISA: Enzyme Linked Immuno Sorbent Assay
FSH: follicle stimulating hormone
GABA: Gamma-aminobutyric acid
Glu: glutamate
GnRH: Gonadotrophin-releasing hormone
H&E: hematoxylin and eosin
HPLC: High-performance liquid chromatography
HPG: hypothalamus-pituitary-gonad
IGF-1: Insulin growth factor-1
IOD: integral optical density
Kiss1r: Kisspeptin 1 receptor
KNDy: kisspeptin−/ neurokinin B−/ dynorphin
LH: luteinizing hormone
ME: median eminence
NGF: nerve growth factor
NKB: neurkinin B
PCOS: Polycystic ovary syndrome
PFA: paraformaldehyde
