# Correction to: Troxerutin protects against DHT-induced polycystic ovary syndrome in rats

**DOI:** 10.1186/s13048-020-00733-5

**Published:** 2020-11-12

**Authors:** Zixuan Gao, Xiaochen Ma, Jing Liu, Yuhang Ge, Lei Wang, Ping Fu, Zhian Liu, Ruiqin Yao, Xiaonan Yan

**Affiliations:** 1 Department of Histology and Embryology, Xuzhou Medical University, Xuzhou, 221009 PR China; 2 Department of Cell Biology and Neurobiology, Xuzhou Key Laboratory of Neurobiology, Jiangsu Key Laboratory of New Drug Research and Clinical Pharmacy, Xuzhou Medical University, 209 Tongshan Road, Xuzhou, 221009 PR China; 3 Clinical Center of Reproductive Medicine, Xuzhou Central Hospital, 199 Jiefang South Road, Xuzhou, 221000 PR China; 4 Department of Human Anatomy, Xuzhou Medical University, Xuzhou, 221009 PR China

**Correction to: J Ovarian Res 13, 106 (2020)**

10.1186/s13048-020-00701-z

Following publication of the original article [1], Figs. 1, 3 and 5 were incorrect. The published original version should be replaced with the updated version.

The original article [1] has been corrected.
